# Social Cognition in Patients with Early-Onset Parkinson's Disease

**DOI:** 10.1155/2021/8852087

**Published:** 2021-01-07

**Authors:** Ana Natalia Seubert-Ravelo, Ma Guillermina Yáñez-Téllez, María Lizbeth Lazo-Barriga, Alejandra Calderón Vallejo, Carlos Eduardo Martínez-Cortés, Adela Hernández-Galván

**Affiliations:** ^1^Neuroscience Department, Universidad Nacional Autónoma de México, Facultad de Estudios Superiores Iztacala, México City, Mexico; ^2^Servicio de Neurología, Hospital de Especialidades Centro Médico Nacional Siglo XXI, México City, Mexico; ^3^Centro de Investigación Transdisciplinar en Psicología, Universidad Autónoma del Estado de Morelos, Cuernavaca, Mexico

## Abstract

Social cognition (SC) deficits have been linked to Parkinson's disease (PD) but have been less well researched than general cognitive processes, especially in early-onset PD (EOPD), despite this population often having greater social and family demands. Most studies focus on recognition of facial emotion, theory of mind (ToM), and decision-making domains, with limited research reporting on social reasoning. The main objective of this work was to compare SC ability across four domains: emotional processing, social reasoning, ToM, and decision-making between patients with EOPD and healthy controls. Twenty-five nondemented patients with EOPD and 25 controls matched for sex, age, and educational level were enrolled. A battery that included six SC tests was administered to all study participants; a decision-making scale was completed by participants' partners. Statistically significant differences were found between patients with EOPD and controls in all subtests across the four SC domains studied. The EOPD group demonstrated worse performance on all tasks, with large effect sizes. Differences remained significant after adjusting for Montreal Cognitive Assessment (MoCA) test scores for all SC subtests except the decision-making scale and the Iowa gambling task. No significant correlations between SC and other clinical PD variables were found. Our study shows that patients with EOPD perform significantly below controls in multiple SC domains affecting recognition of facial emotion, social reasoning, ToM, and decision-making. Only decision-making seems to be mediated by overall cognitive ability. The confounding or contributing effect of other clinical PD variables should be studied further.

## 1. Introduction

Parkinson's disease (PD) is a common neurodegenerative disorder with several cardinal motor symptoms, including bradykinesia, tremor at rest, and rigidity. The estimated global prevalence of PD more than doubled (from 2.5 to 6.1 million) between 1990 and 2016, and experts estimate that it could reach 12 million by 2040 [1]. Although early-onset PD (EOPD; disease onset ≤50 years) reportedly represents a minority of PD cases, age at onset seems to be younger in some clinical populations, including in Mexico [2]. In recent years, nonmotor symptoms, including cognitive impairment and psychiatric disorders, have received attention due to their effect on everyday functioning and quality of life; for this reason, an expert panel recently highlighted the need to address knowledge gaps in cognition-related research [3]. Studies of general cognition in PD populations have led to clearer characterization of cognitive phenotypes as well as estimates of the prevalence rates of PD-related mild cognitive impairment and dementia, even in the EOPD population [4]. Nevertheless, one aspect of cognition that has not been fully explored is social cognition (SC), especially in EOPD.

SC is a complex construct that includes a set of neurocognitive processes underlying the ability to recognize, manipulate, and behave with respect to socially relevant information [5]. It entails a variety of skills, ranging from perceiving and decoding social information and drawing inferences regarding others' mental states to making decisions consistent with social norms and the welfare of others [6]. Although there is no clear consensus as to which abilities integrate the construct of SC, several authors [6–9] suggest four similar aspects of SC, related with brain structures that compose the social brain. First *emotional processing* refers to mental processes that evaluate emotionally relevant information that evokes a bodily emotional state as well as additional changes in mental state [9]. It is considered within the social perception domain of SC, which is deemed as a basic prerequisite of SC [6]. Emotional processing includes emotion identification and comprehension abilities as well as emotion expression [10]. Specifically, facial expressions are considered to represent the most effective means for emotional communication [6]. Second, *social reasoning* refers to the ability to make inferences and deductions in social contexts, which in turn allows individuals to generate problem-solving alternatives, anticipate consequences, and emit judgements within a social context. It requires previous knowledge or information about situations, actors, action options, and possible action results when considered in social contexts [7, 11]. Third, *theory of mind* (ToM) refers to the ability to attribute mental states to oneself or another person [12]. In ToM, research has demonstrated a distinction between cognitive ToM (attribution of mental states) and affective ToM (attribution of emotional states) [6]. Fourth, *decision-making* implicates the assessment of the potential future results of several options through a cost-benefit analysis that ends with the selection of a given solution and its implementation in real life [11]. Impairment of SC has been consistently linked to functional disability, unemployment, poorer quality of life, mental health problems, and impaired social relationships [13–18], all of which are common in the PD population. Moreover, the inability or difficulty in maintaining social relationships and the associated social isolation has been linked to greater mortality and is considered a risk factor for cognitive deterioration [19, 20].

According to several reviews of SC studies in the PD population published between 2007 and 2017, research on SC in PD has focused on ToM, decision-making, and ability to recognize facial emotion and has assessed patients aged 60–71 years [15, 21, 22]. More recent studies have centered on the same SC domains [23, 24]. Their findings demonstrate consistent deficits in ToM, especially cognitive ToM [23, 25, 26], although a growing body of evidence shows that affective ToM is also compromised [25, 27, 28]. Furthermore, deficits in ToM appear to be present even in the early stages of the disease [29] and worsen with disease progression [28] and are not associated with dopamine-based therapy [26]. Although no relationships between SC deficits and general neurocognition have been found [30, 31], there have been reports of a link between ToM and executive functions, including working memory, cognitive flexibility, and inhibition [23, 27].

While decision-making has been less well studied, the evidence suggests that it is also consistently affected in nondemented patients with PD from the early stages of the disease [31, 32] and that it may be related to ToM ability [33].

Regarding recognition of facial emotion, an impaired capacity to recognize anger, disgust, and fear has been repeatedly reported [15], and difficulty with sadness and surprise has also been found [34, 35]. As in other areas of SC, impaired recognition of facial emotion seems to be present from the early stages, and lack of dopamine replacement therapy appears to be related to worse performance on this ability [36]; an association with age and age at disease onset has also been reported [35]. To our knowledge, only one study has addressed abilities within the social reasoning domain in the PD population [37], in which differences were found in social problem-solving ability between patients with PD and controls only when mild cognitive impairment (PD-MCI) was present.

Although some studies have included a minority of patients with EOPD in their samples [27, 33, 36, 38], SC has not been specifically studied in EOPD despite multiple reports indicating that this subgroup of patients perceives their quality of life and emotional wellbeing to be worse overall independent of depression status [39, 40], report less satisfactory marital and family relationships and social life, and perceive more stigmatization [41]; these aspects could all be related to perception, processing, and responding to social stimuli. In addition, certain motor [42, 43], cognitive [4, 42–44], neurobiological [45, 46], and genetic [43] differences have been reported between early- and late-onset PD patients; thus, it is possible that differences in SC ability are also present. Furthermore, given that SC ability can be influenced by normal aging [47], studying social cognitive ability in patients with EOPD allows the assessment of younger participants and could contribute to an understanding of the effect of PD pathology on SC ability without the confounding effect of age-related variables. The main objective of this study was to compare SC ability across four SC domains: emotional processing, social reasoning, ToM, and decision-making between patients with EOPD and controls matched for age and education. We were also interested in exploring the effect of general cognitive ability on SC performance. Finally, we tested the association between SC performance and several clinical variables (age, disease duration, and levodopa-equivalent daily doses (LEDDs)) in the EOPD group.

## 2. Materials and Methods

### 2.1. Participants

The study participants included 25 nondemented patients with early-onset PD (onset of motor symptoms before the age of 50 years), recruited at the Movement Disorders Clinic of the Specialties' Hospital in Centro Médico Nacional Siglo XXI in Mexico City, and 25 healthy nonconsanguineous controls matched for age and education. Age at onset for EOPD has not been strictly defined and varies between studies although the classification is conventionally used in cases with an onset of motor symptoms before the age of 40–50 years [43]. Because there are no reports indicating significant differences in cognitive ability between different cutoffs within this range, we defined EOPD as onset of motor symptoms before the age of 50 years. The control group consisted of patients' spouses (four), nonconsanguineous family members (two), and voluntary participants (19).

Given that most studies state that SC ability seems to be independent of general cognitive ability [30, 31] and because mild cognitive changes are present in up to 25–52% of the PD population [48], we decided to include EOPD participants with a broad range of cognitive ability, to be able to represent the patient population that a clinician would encounter routinely, only excluding those with major cognitive changes.

PD was diagnosed according to the UK Brain Bank Criteria [49]. The study exclusion criteria included major cognitive dysfunction (i.e., Montreal Cognitive Assessment (MoCA) score <21, suggestive of dementia), major depression according to the DSM 5 criteria, comorbid neurological conditions, significant uncorrected visual or hearing impairment, and limited education (<6 years).

All participants agreed to participate in the study and provided written informed consent on a protocol that was evaluated and approved by the hospital's Research and Ethics Committees (registration no. R-2018-3601-047).

After accepting to participate, patients and controls underwent a semistructured interview performed by a clinical neuropsychologist to rule out major depressive disorder and check for other exclusion criteria, as well as a general cognitive screening using the MoCA test [50]. Participants who cleared all exclusion criteria underwent SC assessment in one session of approximately 60 minutes. All patients were assessed in the ON pharmacological condition.

### 2.2. Assessment Instruments

#### 2.2.1. Clinical Evaluation

Patients were classified according to Hoehn–Yahr (H&Y) stage [51] by a movement disorder specialist. Disease duration was calculated as years since onset of cardinal motor symptoms. LEDDs were calculated according to the standard formula [52].

#### 2.2.2. Assessment of Social Cognition

Two instruments were used to assess the SC domains of emotional processing, social reasoning, ToM, and decision-making [6–9, 53].

#### 2.2.3. Social Cognition Battery (COGSOC)

**Figure 1:**
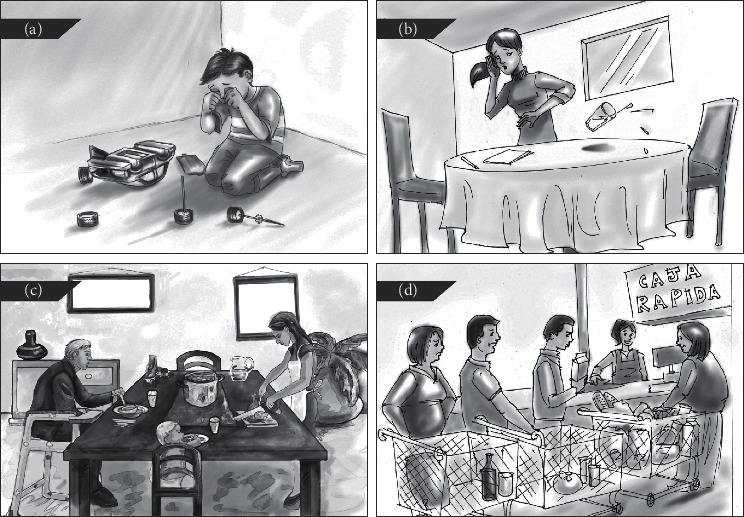
Examples of the COGSOC illustrations used in the causal relationships comprehension, absurdity identification, and social judgment ability subtests. The illustrations presented to patients are in color. (a) Causal relationships comprehension part A-causes: participants are asked “what has most probably happened immediately before this scene?” (b) Causal relationships comprehension part B-consequences: participants are asked “what has most probably happened immediately after this scene?” (c) Absurdity identification: participants are requested to find everything they consider wrong, nonsensical, or absurd. (d) Social judgment: in this item, participants were told “this is a fast cashier line and the lady at the front of the line has more items than that are permitted in her cart, what is the best course of action for the people in line?”

The COGSOC battery [10, 54] was created to provide a clinical instrument for assessment of SC in adults with a range of neurodegenerative and neuropsychiatric conditions. This instrument comprises seven subtests: five original subtests including two causal relationships comprehension tasks (causes and consequences), a visual absurdities identification task, a social judgment ability task, and a decision-making scale completed by caregivers and two known SC paradigms: denomination of Ekman's Pictures of Facial Affect (POFA) and a physical adaptation of the Iowa gambling task (IGT) according to the specifications of Bechara et al. [53]. The battery assesses three SC domains (i.e., emotional processing, social reasoning, and decision-making). The reliability coefficient (*α*) for the battery is 0.9, and for the subtests, it ranges between 0.7 and 0.9. The COGSOC construct validity was analyzed through exploratory factor analysis, which demonstrated that causal relationships comprehension (causes and consequences), visual absurdities identification, and social judgment tasks were grouped as one factor (social reasoning); POFA remained alone as a factor (emotional processing), and finally, the IGT and the decision-making scale was grouped in a third factor (decision-making). The battery was created for use in Mexican adult population and its psychometric properties, including difficulty level, discrimination capacity, and reliability of each item, and subtest has been tested [54].  Table 1 provides further information on the purpose and characteristics of the battery's subtests.  Figure 1 provides examples of the illustrations used in the causal relationships comprehension, visual absurdities identification, and social judgment ability subtests. The subtests are purposely designed to present most stimuli through thematic illustrations, reducing the load on short-term and working memory, which is deemed important when assessing PD population.

#### 2.2.4. “Reading the Mind in the Eyes” Test

The revised version of the “Reading the Mind in the Eyes” (RME) test in Spanish [58, 59] was used to assess ToM. The instrument was created to assess subtle changes in ToM capacity in adults and is considered an advanced ToM test. It has been used widely to measure affective ToM in the PD population [28, 61, 62] because it requires inference of what a person is feeling rather than the person's beliefs or motivations.

Although the POFA and RME tests could seem similar, it is important to consider that the POFA test measures the ability to perceive basic emotions, which are considered universal, are biologically determined, and can be automatically appraised or perceived [55]. In contrast, emotions included in the RME test are secondary or “high-order” emotions. To recognize secondary emotions, one requires cognitive elaboration of a social context and an inference of what the person is feeling. These secondary emotions arise from subtle combination of basic emotions and are considered “complex mental states” [58].

### 2.3. Statistical Analysis

An independent-sample *t*-test was used to compare the results for all the SC subtests between the study groups. Normality for data distribution was assessed graphically. Homogeneity of variance was tested with Levene's test, and the appropriate *t*-test was used. Effect sizes were calculated using Cohen's *d*. To adjust for the effect of general cognitive ability (MoCA scores) on SC performance, *t*-tests were followed by a one-way analysis of covariance (ANCOVA). The assumptions of linearity, homogeneity of regression slopes, normality, and homogeneity of variance were all met for the ANCOVA. The raw scores were compared because the groups were matched for age and educational level. A *p* value <0.05 was considered significant. A Bonferroni correction (*p* < 0.006) was applied to lower the probability of type 1 errors due to multiple comparisons.

We used Fisher's exact test to determine whether or not there was a significant difference in the proportions of patients and controls who incorrectly identified each of the six emotions in the POFA subtest. For the remaining subtests, a description of the types of errors committed by participants in each group and their frequency is provided.

Pearson correlations were used to test if clinical variables (age, disease duration, and LEDDs) were associated with SC performance in the patient group and were considered significant at *p* < 0.05. A Bonferroni correction was used to lower the probability of type 1 error (*p* < 0.002).

All statistical analyses were performed using SPSS version 24 (IBM Corp., Armonk, NY, USA).

## 3. Results

There was no between-group difference in age, educational level, or sex distribution. As expected, the EOPD group had significantly lower MoCA scores than the control group (Table 2). The EOPD group had a mean age at disease onset of 45.3 ± 4.1 years, a mean disease duration of 10.6 ± 4.2 years, and mean LEDDs of 1320.5 ± 528.2. Fifty-six percent of patients was classified as H&Y stage 2, 32% as stage 3, and 12% as stage 4. The mean H&Y stage was 2.6 ± 0.7.

### 3.1. Group Differences in SC Performance

An independent-sample *t*-test was used to determine if there were any between-group differences in performance across the eight subtests measuring four SC domains: emotional processing, social reasoning, ToM, and decision-making assessed by the COGSOC battery and the RME test. The patients with EOPD demonstrated worse performance in all subtests measuring the four assessed SC domains. All between-group differences remained significant even after Bonferroni correction (*p* < 0.006). The effect sizes were large for all comparisons (Table 3). Moreover, all differences remained significant after adjusting the effect of general cognitive ability, except for the decision-making scale; differences in performance on the IGT subtest, also within the decision-making domain, did not remain significant either after Bonferroni correction when controlling for general cognitive ability (Table 3).

### 3.2. Emotional Processing

The EOPD group had a significantly higher misidentification rate when presented with a neutral face (76% vs. 8% of controls; *p* > 0.001), a face expressing happiness (36% vs. 0%; *p*=0.002), and a face expressing surprise (32% vs. 18%; *p*=0.023; Fisher's exact test). The EOPD group tended to label the neutral face as expressing negative emotions such as sadness, boredom, or loneliness. Although the incorrect identification rate was higher in the EOPD sample, no significant differences were found regarding anger, fear, or sadness.

### 3.3. Social Reasoning

The patient group performed significantly worse than controls on all four social reasoning tasks. In both causal relationships comprehension subtests, the patient group tended to provide answers with no immediate causal relationship or answers that reflected an improbable cause/consequence. In the visual absurdity identification subtest, the EOPD group identified on average only 45% of the total absurdities, whereas the control group identified an average of 86%. Finally, in the social judgment task, the patients tended to propose actions that only partially solved the problem or that implied certain risks.

### 3.4. Theory of Mind

In the ToM task, patients with EOPD could not infer the correct mental state in an average of 39% of items on the RME test versus an average of 25% in the control group. Qualitatively, EOPD patients answered more impulsively and persistently inferred mental states related to negative emotions.

### 3.5. Decision-Making

Patients with EOPD tended to choose disadvantageous cards in the IGT: 67% of patients (vs. 16% of controls) made over half of their selections from the high-risk decks (A and B). Controls could identify the high-risk decks within several selections and tended to select few cards from these decks; in contrast, although patients with EOPD could verbally recognize that decks A and B “took their money,” they did not refrain from selecting from those decks. Most patients with EOPD lost all their money before the end of the task (68% vs. 24% of controls). Even though they were permitted to continue until completing 100 selections, patients with EOPD tended to still make disadvantageous choices and demonstrated altered decision-making in everyday life. The most common difficulties reported by caregivers in the decision-making scale included inadequate stewardship of money (84% vs. 12% in controls), poor food choices (56% vs. 4%), and problem-solving difficulties within the family context (76% vs. 32%).

### 3.6. Correlation between Clinical Variables and SC in the EOPD  Group

We found no significant associations between performance on any of the SC subtests and disease duration. Age at assessment was correlated with performance on the absurdity identification and social judgment subtests, and LEDDs were correlated with performance on the POFA and social judgment subtests, but the associations did not remain significant after Bonferroni correction (Table 4).

## 4. Discussion

Our study shows that patients with EOPD perform significantly worse than controls in all SC measures of emotional processing, social reasoning, ToM, and decision-making with large effect sizes for all subtests. The patient group scored 1.5–2 SD below the control group mean for all subtests measuring emotional processing, social reasoning, and ToM and in the lower limit of the 1st SD for decision-making tests. Only performance in the test and the scale measuring decision-making were mediated by general cognitive ability. We found no significant association between SC performance and age at onset, disease duration, or LEDDs.

Our patient sample had a mean age of 56 ± 5.36 years, which is seven to 10 years younger than most PD samples in the reported SC studies. Moran et al. [47] found that normally aging older adults (mean age, 71.8 ± 1.9 years) underperformed on three mentalizing tasks and showed age-related decreases in the BOLD response in the dorsomedial prefrontal cortex, suggesting that even normal aging can influence at least some aspects of SC functioning. Given that most PD studies on SC assess patients with mean ages 60 to 70 years, it is possible that SC deficits commonly reported in such PD populations could represent a synergistic outcome of age-related and PD network dysfunction-related deficits. Despite this assumption, our relatively younger PD sample also showed consistent deficits in all SC domains; these could be attributed to PD itself and not to the aging process.

Given the age at onset of our population, our EOPD sample presented a wider range of disease duration, from four to 19 years, than that of many other PD samples reported in SC studies. Many researchers have shown that SC deficiencies are present in the early stages of PD and worsen with disease progression [28, 38]; nonetheless, in our EOPD sample, we found no significant association between years of disease progression and worsening of SC performance in any domain. Therefore, it is possible that the deterioration in diverse SC domains begins in the early stages of EOPD and remains somewhat stable in the age period of our participants although more studies are needed to confirm such hypothesis.

### 4.1. Emotional Processing

In our study, the emotional processing domain was assessed using the POFA subtest of the COGSOC battery, which includes only one item per emotion. Despite this limitation, we found statistically significant differences in recognition of facial emotion between the EOPD and the control group. Moreover, the effect size of this difference was amongst the largest found. Evidence from studies in several dopamine-related and frontostriatal-related conditions [63–65], medicated and unmedicated patients with PD [36], and functional magnetic resonance imaging studies [66] suggests that brain regions modulated by dopaminergic neurons are involved in recognition of facial emotion, in particular, the so-called “limbic” loop, which involves the anterior cingulate, amygdala, insular and temporal cortices, hippocampus, and ventral striatum and is related to the mesolimbic dopaminergic system.

In our EOPD sample, difficulty recognizing fear and anger was not as common as difficulty identifying positive valence emotions; this finding is not consistent with previous PD studies reporting a specific impairment in ability to recognize negative valence emotions [15, 67, 68]. However, we did observe that almost 70% of patients judged a neutral face as expressing an emotion with a negative valence (sadness, loneliness, or boredom). A review showed that most studies of recognition of facial emotion in PD have not reported performance regarding the identification of neutral faces [68]. Although a recent meta-analysis did report that small deficits exist when perceiving neutral faces [69], the authors do not discuss possible mechanisms underlying attribution of emotional valence to neutral stimuli. Given that our patients confounded neutral faces as expressing only negative valence emotions, it is possible that this result reflects dysfunction with specific amygdala-related and mesolimbic pathway-related circuits underlying the processing of negative emotions [66]. Taking into consideration that perception of emotion is considered a prerequisite for other SC abilities, such as ToM and social reasoning [6], it is possible that perceiving neutral faces as a source of negative emotion could be associated with a specifically dysfunctional and negative bias that further affects posterior processing of social information although this hypothesis remains to be tested. We also found that almost half of the patients presented difficulties in identifying surprise and happiness, which are considered emotions with a positive valence. Although altered perception of surprise and happiness has also been reported in PD, it is found to be less common and severe [68, 69]. One explanation given as to why happiness tends to be the easiest emotion to recognize is that it is the only “true” positive emotion included in POFA; thus, it is less likely that participants will confuse it with similar emotions (e.g., excitement) [69]. Despite this hypothesis, 32% of patients with EOPD and none of the controls were unable to identify happiness. This marked between-group difference in frequency suggests condition-related impairment rather than measurement error. Neural mechanisms underlying recognition of positive emotion are less well understood; nevertheless, a dysfunction of specific amygdala circuits is hypothesized [69]. Another mechanism that could be related to altered recognition of happiness is related to the feedback hypothesis and the embodied simulation theory, which, respectively, state that facial expressions influence emotional experiences via sensory feedback and that mirroring the other's facial emotional expressions (i.e., mimicry) via engagement of the corresponding motor circuits and muscular contractions underpins understanding their meaning [6]. In PD, it has been suggested that disturbed motor processing (hypomimia in particular) can lead to deficits in recognition of emotion [68]. A recent study found a significant decrease in facial mimicry, mainly for joy (vs. anger) [70]; using facial electromyography, the authors found almost no reaction of the orbicularis and zygomaticus in response to happy faces. According to the aforementioned theories, our findings could relate to this diminished facial mimicry in PD.

### 4.2. Social Reasoning

Abilities within the social reasoning domain, that is, the ability to make inferences and deductions in social contexts taking into consideration cause-effect relationships, identification of incongruence within a social context, and generation of solutions to problems within the personal or social domain, have rarely been tested in the PD population. To our knowledge, only one study by Anderson et al. specifically addressed the ability to solve problems in a social context [37]. In their study, there were two tasks that required participants to generate and select potentially appropriate solutions in response to hypothetical scenarios depicting everyday problems. Although the studied samples were older at time of assessment than in our study, Anderson et al. only found social problem-solving deficits in patients with PD-MCI. They interpreted this finding as suggesting that the core pathophysiology of PD itself is not responsible for difficulties in social problem-solving and that the latter only occurs in the context of more general cognitive difficulties. Even though our task differed in some respects to the ones used in the aforementioned study, our EOPD sample also demonstrated significantly worse social judgment ability in comparison with controls: they tended to propose solutions that were only partially efficient or that implied risk. Nonetheless, in our sample, differences remained significant even after adjusting for general cognitive ability. Considering that patients with EOPD frequently demonstrate executive dysfunction [4] that has been linked to dysfunction of frontostriatal circuits, specifically the “associative loop” involving the dorsolateral prefrontal cortex, an alternative explanation could be that social judgment abilities depend on networks that also involve the dorsolateral prefrontal cortex subserving SC domains such as social perception [69] rather than being directly dependent on general cognitive dysfunction.

Within the social reasoning domain, our EOPD sample also demonstrated significant differences from controls regarding appropriate inference of causal relationships and identification of visual absurdities (i.e., nonsensical information) in the social context. Differences in the absurdity identification task showed the largest effect size; patients could, in average, identify only around half of the absurdities as controls did. To our knowledge, the ability to identify social absurdity has not been previously investigated in PD. However, because it is an ability that depends not only on social knowledge but also on a correct visual exploration and attention, it is possible that the deficits we found are influenced by more basic processes. Several studies have found visual alterations in PD, including smaller saccades and defective visual-spatial disembedding that affects visual scanning and complex visual perception [71–73]. Moreover, in a previous study of general neurocognition in EOPD, our group found that over 60% of patients had a score below one standard deviation on a superimposed image discrimination task [4], suggesting difficulties when analyzing complex visual stimuli that could affect the visual analysis of complex social scenarios. Regardless of the cause, deficiencies when analyzing visual social information could have a negative effect on social functioning; nonetheless, it remains to be tested whether or not altered identification of visual nonsensical information in the social context is independent or secondary to such visuomotor difficulties.

### 4.3. Theory of Mind

Our study also found significantly worse ToM ability in patients with EOPD compared to controls, with a large effect size, consistent with previous reports of dysfunction in ToM ability in PD [25, 30]. In contrast, our study contradicts two studies by Péron et al. [38, 74] that report no differences between PD patients with a mean age similar to our EOPD sample (mean age at assessment of 56 ± 7.8 and 53 ± 8.5) and healthy controls in ToM ability measured by the RME test. Although the studies by Peron et al. do not specify participants' age at disease onset, pondering the mean ages and mean disease durations (10.2 ± 4.9 and 10.5 ± 3.6 years, respectively), it can be assumed that at least some participants were EOPD patients. A key difference between our study and the aforementioned studies by Peron et al. [38, 74] is that although described by the authors as advanced PD, their samples had mean H&Y stages of 1.2 ± 0.6 and 1.3± vs. 2.6 ± 0.7 in our sample and 32% of patients being in H&Y 3 and 12% in H&Y 4.

Although not all studies have reported affective ToM deficiencies in advanced PD [38], Poletti et al. [28] and Romosan et al. [75] reported worsening affective ToM ability as the disease progresses. Although we did not find a significant correlation between ToM performance and disease duration, almost half of our sample consisted of patients in H&Y stages 3 and 4, which are considered moderate and advanced stages of PD; this could help to explain our findings.

Several studies have linked performance of PD patients on affective ToM tasks with other cognitive deficiencies, including visuospatial ability, inhibition, cognitive flexibility, and working memory [23, 27, 75]. Although we found that differences in ToM performance between EOPD and controls were not mediated by overall cognitive ability, deficiencies in specific cognitive domains could have impacted performance in our patients.

Previous studies have reported an association between affective ToM ability and quality of life in PD [25]. Given that we found significant differences in ToM ability between EOPD patients and controls and that this PD subgroup tends to report significant alteration of their quality of life [39, 40], the association of both variables in EOPD should be assessed.

### 4.4. Decision-Making

We found that patients with EOPD not only performed worse than controls when making decisions under ambiguity in an experimental paradigm but also in real life (according to caregiver reports). Sixty-eight percent of our patients, almost triple the number of controls, lost all their money in the IGT despite consciously identifying the high-risk decks, and a clear tendency towards risky choices was observed in the patient group. Similarly, 84% of patients (vs. 12% of controls) managed their money inadequately, which was the main decision-making problem reported by caregivers in real life.

Decision-making is a complex ability that requires both cold cognitive and emotional processing. In two studies analyzing PD samples older than that of our study (mean age 69.9 ± 8.9 and 60.73 ± 11.79 vs. 56.2 ± 5.36), researchers found significant differences in decision-making under ambiguity and a tendency towards risky choices even in participants with normal general cognitive ability (MMSE matched to healthy controls) [32, 33]. Such differences have been linked to dysfunction of the limbic loop involving the amygdala, orbitofrontal cortex, ventromedial prefrontal cortex, cingulate cortex, and the ventral striatum, which is known to be affected in PD [33]. In contrast, we found that poor performance of EOPD patients in both measures within the decision-making domain, including the same tendency towards risky choices described in the aforementioned studies, was mediated by general cognitive ability. Such results suggest that, in our EOPD sample, altered decision-making is probably associated with the combined effect of a dysfunctional limbic loop and changes in general cognitive ability.

Moreover, our finding that patients with EOPD also scored poorly on ToM ability is consistent with previous reports showing that deficient affective ToM ability in PD could negatively influence decision-making [31] and that both processes could share similar neural mechanisms [33]. Emotional arousal is also implicated in decision-making. In another study in the PD population, low skin conductance responses, a measure of emotional arousal before making decisions and after receiving a reward or punishment, were found to accompany worse IGT performance [32]. The investigators linked this finding to dysfunction of the amygdala, which is known to be involved in risk evaluation. Dysfunction of the amygdala, diminished emotional arousal, and altered risk evaluation would explain why our patients continued choosing from decks A and B in the IGT until losing all their money despite consciously identifying them as the high-risk decks.

### 4.5. Correlation between Clinical Variables and SC in the EOPD  Group

We found no significant correlations between SC ability and other clinical variables, such as age at assessment, disease duration, or LEDDs. Our results are consistent with previous reports indicating a lack of association between ToM [26] and decision-making [32] and dopamine replacement therapy. In the same line, a review on facial emotion recognition in PD also states that although some studies have found better performance in medicated PD patients, many studies do not find a correlation between LEDDs and facial emotion recognition [68]. However, given our small EOPD sample, the absence of significant correlations could also be attributed to lack of statistical power.

### 4.6. Study Limitations

Our study had several additional limitations. First, although our assessment instruments included several paradigms that have been repeatedly used to evaluate SC in neuroscience and PD-specific studies, we used a novel battery that includes original subtests, which limited our ability to make comparisons with the previous literature. In the battery, the POFA subtest only included one item representing each emotion. Nonetheless, the battery has the advantage of being designed for the Mexican population, and its psychometric properties have been tested in said population: an advantage over other SC measurement instruments, which are often criticized for being designed only for research, and not having their psychometric properties tested. However, these instruments allowed us to assess SC domains not previously studied in PD and proved to be useful for distinguishing between patients with PD and controls, which suggests good external validity. Another potential limitation is that even though we excluded patients with major depressive disorder, we did not test the relationship between minor depressive symptoms and SC. Furthermore, we did not test the effect of anxiety on SC abilities. In addition, given assessment time limitations, we did not include other aspects of SC such as cognitive ToM or empathy; we were also not able to perform a detailed neuropsychological assessment and thus used only MoCA for cognitive screening, thereby limiting investigation of the association between specific cognitive processes and SC. Finally, we did not calculate the sample size, which could limit our ability to generalize our findings regarding EOPD. Nonetheless, given the nature of the study population and the complexity of neuropsychological assessment, most studies have included similar sample sizes, even when evaluating the more common late-onset PD.

## 5. Conclusion

In conclusion, our sample of EOPD patients demonstrated a decreased performance in comparison to controls in all the assessed SC domains, affecting the ability to perceive emotions, reason in social contexts, solve social problems, attribute affective states, and make decisions, including in real-life situations. Only decision-making was found to be mediated by general cognitive ability. No significant association between clinical variables and SC domains was found. However, the contribution of these variables or their confounding effects should be further examined. To our knowledge, this is the first report of social cognition ability in EOPD patients, suggesting that SC ability significantly differs from healthy controls in this population. This finding is considered important given the reported link between SC and functional disability, unemployment, and impaired social relationships, which could further impact EOPD patients in a different way than in the usually studied older PD populations.

## Figures and Tables

**Table 1:** Description of the assessment instruments.

SC domain	Test	Subtest	Purpose and characteristics
Emotional processing	COGSOC [10]	POFA	Assesses the ability to identify emotional expressions in faces. Ekman's classical research on emotion expression and comprehension [55] and posterior research by Ekman's group culminated in the development of the POFA materials available for research. Further studies found that emotional expression recognition tasks are sensitive to SC dysfunction (e.g., [56, 57]) and thus are commonly used as part of SC assessment. In the COGSOC battery, the POFA subtest consists of 6 pictures printed in black and white in a half-letter size sheet, one for each basic emotion: anger, fear, happiness, sadness, and surprise plus a neutral expression. Additionally, a different picture representing fear is shown as an example at the beginning of the test. For each picture, the participant has to denominate the perceived emotion. The total subtest score ranges from 0 to 6.

Social reasoning	COGSOC [10]	Causal relationships comprehension A—causes	Assesses the comprehension of cause-effect relationships within a social context. Each part, A and B, consists of eight and six illustrations, respectively, depicting scenes representing simple actions involving a maximum of two characters, printed in color in a half-letter-sized sheet. The participant is asked to verbally provide the most probable, logical, and immediate action that took place before (causes—part A) or after (consequences—part B) the scene. The textual answer is registered for each of the 14 items and then scored according to a 3-point scale: 0 points when the answer has no causal connection with the scene; 1 point when the causal relation is not immediate or is unlikely; and 2 points when the answer reflects a logical, immediate, and probable relation to the scene. The test includes a guide with common answers for each answer level (0–2) to facilitate scoring (similar to the answer scoring guide given in Wechsler scales). The total score for part a ranges 0–16 and for part B 0–12.
		Causal relationships comprehension B—consequences	
		Absurdity identification	Assesses the ability to identify incongruence within a social context and provides information about social knowledge, which is necessary prior to emitting judgements, solving a social problem, or making a decision. It comprises six illustrations, each printed in color in a letter-sized sheet. Each illustration contains a scenario with three to five absurdities that sum a total of 23 items. Participants must observe, without a time limit, each scenario and point out what is absurd, illogical, or incongruent. The total score is the total number of absurdities correctly identified and thus ranges 0–23. It should be noted that participants must search without any verbal or physical cues from the evaluator scene, in which some of the absurdities are not centrally positioned; therefore, this subtest has a higher visual scanning demand in comparison to other subtests in the battery.
		Social judgment ability	Assesses the ability to generate solutions to problems within the personal or social domain. It measures the ability to comprehend, evaluate, and generate a logical, viable, and safe solution to a problem. It estimates social knowledge and reasoning. In the subtest, 11 different social problems are represented visually, each using an illustration printed in color in a letter-sized sheet. Each illustration is accompanied by a verbal statement given by the evaluator, which specifies the problem and states a question. Given that, in some illustrations, more than one character can be involved in the scene, and the complementary question is necessary to inquire about the actions of a specific character. The textual answer is registered for each of the 11 items and then scored according to a 3-point scale: 0 points when the proposed action is inconvenient and illogical or does not solve or further complicates the problem, 1 point when the action partially solves the problem or implies certain risk, and 2 points when the action offers a viable, correct, and safe solution to the problem. The test includes a guide with common answers for each answer level (0–2) to facilitate scoring. The total subtest score ranges from 0 to 22.

ToM	RME test revised [58]		The test assesses the first stage of ToM, at which an attribution of the type of mental state is necessary. It requires a mental state lexicon and semantics of each term; it then involves mapping the term to fragments of facial expressions, that is, matching the eyes in each picture to examples stored in memory and seen in the context of particular mental states. We used the revised version in Spanish [59], using the materials freely available at the autism research centre website. The test is composed of 36 pictures of the eye region of human faces printed in black and white, 19 corresponding to men and 17 to women. The participant must match one of the four words describing a mental state to each of the pictures. If needed, the participant can ask the evaluator for the definition of any of the four possible answers for each picture, according to a “dictionary” provided by the test. One point is given for each correct answer, total score ranges 0–36.

Decision-making	COGSOC [10]	IGT	Widely used for the evaluation of SC, it assesses the implementation of decision-making in real life (i.e., the last stage in the process of problem-solving) [11, 60] under an ambiguous situation. The COGSOC uses the physical version of the IGT described by Bechara et al. [53] with two adaptation that do not alter the task's structure: (1) facsimiles of US dollar bills were substituted for Mexican peso bills without altering the denominations (i.e., 50 dollar bills were substituted for 50 peso bills) or the penalty amounts; (2) following a posterior recommendation of the authors, the number of cards in each deck (A–D) was raised from 40 to 60, given the probability that the cards of certain decks could runout due to perseverative responses, forcing the participant to choose from a nondesired deck. The task ends when the participant has completed 100 selections. The total score of the subtest is the total number of chosen advantageous cards (selections from decks C + D) minus the total number of chosen disadvantageous cards (selections from decks A + B). Scores ≤0 indicate overall disadvantageous decision-making.
		Decision-making scale	The scale aims at assessing decision-making in everyday life using information given by an informant. It takes into consideration that insight might be compromised in a diversity of conditions and thus self-report not be reliable; therefore, information conveyed by an informant is considered more objective. The scale uses a 5-point Likert format and is composed of 18 items that evaluate six indexes of daily decision-making: general (3 items), home-security (4 items), finances (2 items), shopping (2 items), interpersonal relations (2 items), and self-care (5 items). The total score ranges 18–90, with higher scores representing better decision-making ability.

COGSOC, social cognition battery; SC, social cognition; POFA, Pictures of Facial Affect; ToM, theory of mind; IGT, Iowa gambling task; RME, reading the mind in the eyes.

**Table 2:** Demographic characteristics and general cognitive ability of the EOPD (*n* = 25) and control (*n* = 25) groups.

	EOPD		*t* (*p*)	*χ* ^2^ (*p*)
	Mean (SD)	Control		
Age (years)	56.2 (5.36)	55.3 (7.46)	0.501 (0.619)	
Education (years)	11.4 (2.66)	11.5 (2.62)	−0.107 (0.915)	
MoCA	25.6 (1.47)	27.4 (1.41)	−4.41 (0.001)	
Sex, male (%)	72%	68%		0.095 (0.758)

EOPD, early-onset Parkinson's disease; MoCA, Montreal Cognitive Assessment; SD, standard deviation.

**Table 3:** Comparison of performance in SC between the EOPD (*n* = 25) and control (*n* = 25) groups before and after adjusting for overall cognitive ability.

SC domain	Subtest	EOPD		*t* (*p*)	*d*	EOPD		SE	Adjusted *F* (*p*)	Partial eta squared
		Mean (SD)	Control			Adjusted mean	Control			
Emotional processing	POFA	3.56 (0.92)	5.04 (0.84)	−5.95 (<0.001)^*∗*^	1.68	3.74	4.86	0.18	15.77 (<0.001)^*∗*^	0.251
Social reasoning	Causal relationships comprehension A—causes	9.60 (2.75)	13.56 (1.64)	−6.18 (<0.001)^*∗*^	1.75	10.07	13.09	0.47	17.40 (<0.001)^*∗*^	0.270
	Causal relationships comprehension B—consequences	8.68 (1.49)	10.52 (1.23)	−4.76 (<0.001)^*∗*^	1.34	8.76	10.44	0.30	13.35 (0.001)^*∗*^	0.221
	Absurdity identification	10.60 (5.28)	19.76 (3.22)	−7.41 (<0.001)^*∗*^	2.09	11.13	19.23	0.95	31.16 (<0.001)^*∗*^	0.399
	Social judgment	15.24 (2.52)	18.92 (2.52)	−5.17 (<0.001)^*∗*^	1.46	15.46	18.70	0.55	14.74 (<0.001)^*∗*^	0.239
ToM	RME test	21.36 (4.44)	26.60 (3.61)	−4.58 (<0.001)^*∗*^	1.29	21.95	26.01	0.87	9.28 (0.004)^*∗*^	0.165
Decision-making	IGT	−12.48 (16)	8.24 (21.39)	−3.88 (<0.001)^*∗*^	1.10	−10.84	6.60	4.15	7.56 (0.008)	0.139
	Decision-making scale	65.68 (9.94)	75.16 (10.53)	−3.27 (0.002)^*∗*^	0.93	67.98	72.86	2.12	2.28 (0.138)	0.046

EOPD, early-onset Parkinson's disease; IGT, Iowa gambling task; POFA, Pictures of Facial Affect; RME, reading the mind in the eyes; SC, social cognition; ToM, theory of mind; SD, standard deviation; SE, standard error. ^*∗*^Significant differences after Bonferroni correction (*p* < 0.006).

**Table 4:** Pearson correlations between age, years of disease progression, levodopa-equivalent daily doses, and SC variables in the EOPD group (*n* = 25).

	POFA	Causal relationships comprehension A—causes	Causal relationships comprehension B—consequences	Absurdity identification	Social judgment	RME test	IGT	Decision-making scale
Age (years)	−.116 (0.422)	−0.149 (0.301)	−0.070 (0.630)	−0.289 (0.042)^*∗*^	−.328 (0.020)^*∗*^	−0.094 (0.517)	−0.114 (0.429)	−0.177 (0.219)
Disease progression (years)	−.064 (0.759)	−0.053 (0.803)	0.260 (0.209)	−0.202 (0.332)	0.123 (0.559)	−0.156 (0.457)	−0.148 (0.481)	−0.025 (0.906)
LEDDs	0.442 (0.027)^*∗*^	−0.265 (0.201)	−0.347 (0.089)	0.176 (0.401)	−0.458 (0.021)^*∗*^	−0.394 (0.051)	0.247 (0.234)	−0.143 (0.496)

EOPD, early-onset Parkinson's disease; IGT, Iowa gambling task; LEDDs, levodopa-equivalent daily doses; POFA, Pictures of Facial Affect; RME, reading the mind in the eyes; SC, social cognition; ToM, theory of mind. *p* values are provided in parenthesis ^*∗*^*p* < 0.05. No correlations remained significant after Bonferroni correction (*p* < 0.002).

## Data Availability

The neuropsychological and clinical data used to support the findings of this study are available from the corresponding author upon request.
